# Work-related psychosocial demands related to work organization in small sized companies (SMEs) providing health-oriented services in Germany – a qualitative analysis

**DOI:** 10.1186/s12889-022-12700-4

**Published:** 2022-02-24

**Authors:** Anke Wagner, Elena Tsarouha, Eylem Ög, Christine Preiser, Monika A. Rieger, Esther Rind

**Affiliations:** Institute of Occupational and Social Medicine and Health Services Research, University Hospital of Tübingen, Wilhelmstraße 27, 72074 Tübingen, Germany

**Keywords:** Small-sized enterprises, Psychosocial demands, Work-related demands, Organization of work, Qualitative methods, Qualitative content analysis, Germany

## Abstract

**Background:**

Micro, small and medium-sized enterprises (SMEs) represent the majority of businesses in the EU. Little is known about psychosocial demands faced by company owners, managers, and employees in SMEs, especially in the health and service sector. The current study aimed to identify which psychosocial demands related to work organization are reported by managers and employees in the health and service sector, and if managers and employees differ in their perspective on these psychosocial demands.

**Methods:**

We conducted nine single interviews and two focus group discussions with seven company owners and managers as well as eleven employees from six different German companies between January and February 2020. The psychosocial factors of the psychosocial risk assessment of the Joint German Occupational Safety and Health Strategy (GDA) served as a framework for data collection and analysis. The interview material was analysed using Mayring’s method of qualitative content analysis.

**Results:**

We identified four prevailing work-related psychosocial demands related to work organization among managers and employees: (1) possibilities and time for recovery after work, (2) communication and cooperation, (3) work intensity, and (4) interruptions, and prioritization. According to the managers, they were confronted with a lack of possibilities and time for recovery after work. They report issues related to inadequate communication and cooperation affecting the entire company team, and also face high work intensity and frequent interruptions and prioritization. Employees reported a clearer division between work and private life. However, they also face periods of high work intensity, frequent interruptions and the need for prioritization.

**Conclusion:**

Managers and employees in SMEs in the health and service sector would benefit from evidence-based and evaluated tailored interventions and approaches for improved work organization. Further studies are needed to support managers and employees in SMEs in the health and service sector in facing and handling work-related psychosocial demands like lack of possibilities and time for recovery after work, high work intensity or frequent interruptions.

**Supplementary Information:**

The online version contains supplementary material available at 10.1186/s12889-022-12700-4.

## Background

Perceived work-related stress has increased substantially in recent years. The stress and strain concept is well suited to describe causes and consequences of work-related stress [1, 2]. According to Rohmert, work-related stress is affected by work content and task, work environment (e.g., climate, noise, lighting), and social or psychological factors (e.g., leadership, personal relations, communication and cooperation) [1, 2]. However, according to the stress and strain concept, stress can lead to different levels of strain including positive or negative effects depending on an individual's abilities and skills [1, 3].

Several studies have confirmed that constantly high levels of work-related psychological stress may have harmful effects on people’s health; well-known consequences are burnout, depression, anxiety, nervousness, fatigue, continuous tiredness, headaches, a lack of concentration, a lack of energy, digestive problems, sleep problems and physical illnesses, for example musculoskeletal or cardiovascular problems or illnesses resulting from a weakened immune system [4, 5]. Other negative outcomes of work-related psychological stress were absence of work, reduced productivity, reduced quality of products and services, and reduced employee engagement and motivation [5]. In Germany, the recent publication “Stress Report “ revealed that in particular employees in health professions are exposed to increased psychological work-related stress [6].

According to EU regulations and national safety laws, employers are required to perform a risk assessment in each workplace in order to identify work hazards and derive necessary occupational health and safety interventions for their employees [7]. The risk assessment also includes the assessment of psychosocial factors that may result in work-related stress and strain and respective preventive measures [8]. Based on established theories and models (e.g., the job demand-control model [9], the demand-control-support-model [10], the effort-reward-imbalance-model [11], the concept of organizational justice [12, 13], and the Job Demands-Resources (JD–R) model) [14]) describing the development of work-related psychosocial stress and strain, the Joint German Occupational Safety and Health Strategy (GDA) provides a framework classifying psychosocial factors into the following structure: “Work content and task”, “Organization of work”, “Social relations”, “Working environment”, and “New forms of work” [3, 15]. For every factor, positive aspects (i.e. resources) or negative aspects (i.e. stressors) with regard to the employees’ wellbeing can be described as derived from the theories and models mentioned above.

While the (physical, chemical, biological, psychosocial) risk assessment and thus the identification of particular job demands and job resources has found widespread adoption in large companies [16], its implementation in small and medium-sized enterprises (SMEs) is still challenging [17]. Particularly the inclusion of a psychosocial risk assessment is rather inconsistent [18]. Small and medium-sized enterprises (SMEs) represent, however, 99% of all businesses in the EU [19] and, compared to larger companies, have often lower financial and human resources for occupational health and safety. This can lead to a lower priority placed on occupational health and safety [20], especially with regard to preventing or reducing work-related psychosocial stress.

To date, only a few studies have examined work-related psychosocial stress in SMEs. A recently published review provided an overview of the state of knowledge regarding work-related psychosocial demands and stressors in different SMEs using the classification of the GDA [21]. The authors identified 45 studies investigating psychosocial factors at work in different SMEs. Most studies applied a cross-sectional design, and focused on factors concerning organization of work, and work content and task. Frequently examined topics for organization of work and work content and task were work time, work process, communication/cooperation as well as freedom of action, responsibility and emotional demands. Other psychosocial factors of the GDA, such as social relations, working environment and new forms of work have been considered less in studies so far. The authors concluded that more research on psychosocial factors in SMEs is crucially needed, and that other economic sectors besides manufacturing should be considered. A qualitative study by Pavlista et al. revealed that SMEs need in general more support and approaches for dealing with psychosocial stressors at work [22]. The authors interviewed 18 owners and managers from 15 smaller companies in Germany and identified different barriers regarding the implementation of the psychosocial risk assessment [22]. Main identified barriers were stigmatization of mental illness, lacking acceptance of employees, not understanding the necessity of the psychosocial risk assessment, or an inappropriate approach [22]. Other studies also confirmed that smaller companies need more support and an infrastructure for the prevention of psychosocial risks at work [23].

Current research recently identified various work-related psychosocial demands, stressors and resources of general practitioners (GP) and their primary care teams [24–26], including, for example, frequent interruptions, high work intensity, and high noise levels in practice rooms [25]. Based on the findings, a complex intervention for the improvement of job satisfaction and the prevention of work-related stress of GPs and their primary care teams as a model for SMEs was developed and evaluated in Germany [27]. In Australia, the Business in Mind program also investigated psychosocial stress of owners and managers from different SMEs, and employed a complex mental health promotion intervention [28–30], which was able to contribute to a successful mental health promotion and a reduction of symptoms of psychological distress among owners and managers from SMEs [29].

While work-related psychosocial stress in SME settings in the primary care sector has been well documented, e.g., [31–37], there are currently, to the best of our knowledge, no studies that capture work-related stress from the related health and service sector. According to SME statistics, the health and service sector comprises the following business types: optician, hearing aid company, orthopaedic technician, orthopaedic shoemaker, dental technician, pharmacy and cosmetic studio [38]. Managers and employees of these companies are exposed to increasing work-related demands, for instance due to urgent orders and deadlines, frequent customer contact, and dependencies on external suppliers. In our study, we therefore aim to get a better understanding of work-related psychosocial demands of managers and their employees in SMEs in the health and service sector with reference to the established framework of the GDA and the mentioned psychosocial factors within. We used in our study the definition from the J-D R model to define job demands. According to this definition, job demands comprise, besides physical aspects, all psychological, social or organizational aspects at work that were associated with psychological (cognitive and emotional) effort and certain consequences [14]. We aimed to explore which work-related psychosocial demands are reported by managers and employees in the health and service sector. We discovered in the course of our study, that aspects of work organization prevailed in our material and were closely interconnected to several other work-related psychosocial demands. So, we focused for this publication on aspects of work organization and pursued the following research question:

- Which psychosocial demands related to work organization are reported by managers and employees in the health and service sector, and do they differ in their perspective on these psychosocial demands?

Answering this research question helps to discuss possible implications for handling work-related psychosocial demands related to work organization in SMEs in the health and service sector.

## Methods

### Study design

We applied a qualitative research design [39], since it enables an in-depth and comprehensive analysis of work-related stress experienced by entire teams. We planned semi-structured interviews with company owners and executive managers as well as focus group discussions with employees from different enterprises in the health and service sector to identify work-related psychosocial demands relevant from the company owner/manager as well as the employee perspective. Initially, we planned to conduct a participatory observation of team meetings to triangulate different methods to continue our recent study addressing psychosocial demands in general practices [25, 26]. During the recruitment process, it became evident that none of the included companies conducted regular team meetings. Therefore, we decided to drop the participatory observation. To avoid issues concerned with hierarchy and explore topics around leadership, we planned to interview the management staff separately from other team members. The study followed the consolidated criteria for reporting in qualitative research (COREQ) [40] (Supplementary Material 1). Ethical approval was obtained from the responsible Ethics Committee of the Medical Faculty and University Hospital of Tübingen (reference number: 585/2019BO2).

### Recruitment

A sample of enterprises in the health and service sector in Baden-Wuerttemberg (South of Germany) was selected via a comprehensive internet search. Based on SME statistics [38], we aimed to include all five sectors: optician, hearing aid company, orthopaedic technician, orthopaedic shoemaker, dental technician. We further wanted to include a pharmacy as well as a cosmetic studio providing health-oriented services such as medical hand and foot care. ER and EÖ approached the enterprises in person, shortly introducing the study to a member of available staff. They left a study invitation for the company owner, an overview of the study including information about data protection, as well as individual forms of consent for all members of staff emphasizing that the study was voluntary. If the company was interested to participate, they were asked to return a feedback form via email or fax. None of the companies had an employee organization that had to agree to conduct the study. If a business did not reply or did not wish to participate, another enterprise of the same type was chosen from the convenience sample. Overall, ER and EÖ visited and invited 19 companies of which eight companies declined participation for various reasons (e.g., no interest, no time, shortage of staff), and five companies did not respond. Six companies agreed and were included in our study.

### Data collection and study population

To structure the interviews and focus group discussions, the interviewers used an interview guide based on a theoretical framework by the Joint German Occupational Safety and Health Strategy [3] and previous research [24, 25]. The interview guide focused mainly on these topics of the psychosocial risk assessment [3]: (1) Work organization, (2) Work task, and (3) New forms of work. (see Supplementary Material 2). Since we wanted to encourage discussion on these topics, the questions of the interview guide were put in a narrative-generating way and we avoided yes/no questions. The interview guide was previously tested and large parts of it were successfully applied in a study among managers and employees in general practices as another SME setting [24–26].

Data collection took place between January and February 2020 in the respective companies. ER and EÖ conducted five single interviews with the company owners and executive managers (pharmacy, optician and hearing aid company, orthopaedic company including a medical supply store, orthopaedic shoemaker, and dental technology company) and one interview with two business partners (cosmetic studio). For organizational reasons (e.g., available time, number of available staff, number of customers present at the time of the interviews/focus groups), it was only possible to conduct two focus group discussions, one with 5 staff members working at the optician and hearing aid company and the other with 3 staff members of the dental technology company. The employees of the cosmetic studio decided at short notice not to participate (no further reasons given). During the two focus group discussions, it became apparent that participants often agreed on their common views and attitudes. Often, there was a shared approval to individual comments and additional thoughts were added. The shared attitudes toward job demands was probably due to the fact that the employees had been working together for a very long time. Due to the number of available staff, we conducted three single interviews with staff members working in the orthopaedic company, at the shoemaker with a specialisation in orthopaedic mechanics, and the pharmacy. The interviews and focus group discussions took place in the respective companies, and were audio recorded. The average duration of all interviews was 49 min. The duration varied between 36 and 78 min.

Besides the interviews, we took notes to document aspects of the atmosphere during the interviews and focus group discussions providing additional context to inform the subsequent data analysis [41]. All interviewed managers of the enterprises completed a short questionnaire depicting general characteristics of the SME (e.g., number of employees, kind of employment, qualifications of employees). As presented in Table 1, the study population covered the broad range and heterogeneity of SMEs in the health and service sector.

**Table 1:** Overview of company characteristics and the study population

Health-oriented business sector and type of company	Characteristics of managers	Number of employees	Qualification of employees	Work time models	Employment contract
Pharmacy	head of the company: eight years	*n* = 16	completed academic training (*n* = 9)	full time employees (*n* = 2)	permanent contracts (*n* = 14)
No family business	responsible for another branch office		completed vocational trainings (*n* = 4)	part-time employees (*n* = 14)	temporary contracts (*n* = 2)
	additional executive managers		currently undertaking vocational training (*n* = 3)		
Optician and hearing aid company	head of the company: nine years	*n* = 24	completed academic training (*n* = 2)	full time employees (*n* = 12)	permanent contracts (*n* = 24)
family business	responsible for another branch office		completed vocational trainings (*n* = 10)	part-time employees (*n* = 9)	
	additional executive managers		under vocational training (*n* = 4)	other work models (*n* = 3)	
			other qualification (*n* = 5)		
Orthopaedic company	head of the company: two years	*n* = 9	completed vocational trainings (*n* = 7)	full time employees (*n* = 4)	permanent contracts (*n* = 9)
family business	another co-manager		under vocational training (*n* = 1) other	part-time employees (*n* = 5)	
			qualification (*n* = 1)		
Orthopaedic shoemaker	head of the company: 34 years	*n* = 2	completed vocational trainings (*n* = 2)	full time employee (*n* = 1)	permanent contracts (*n* = 2)
family business	responsible for another branch office			part-time employee (*n* = 1)	
	another co-manager				
Dental technology company	head of the company: 20 years	*n* = 14	completed vocational trainings (*n* = 10)	full time employees (*n* = 13)	permanent contracts (*n* = 14)
No family business	additional executive managers		under vocational training (*n* = 2)	part-time employees (*n* = 1)	
			other qualification (*n* = 2)		
Cosmetic studio	head of the company: 13 years	*n* = 14	completed vocational training (*n* = 14)	full time employees (*n* = 6)	permanent contracts (*n* = 14)
family business	responsible for another branch office			part-time employees (*n* = 8)	
	another co-manager				

### Data analysis and application of quality criteria

The recorded interviews were transcribed by a professional transcription service using a simplified transcription system [42]. The simplified transcription system was applied considering the transcription rules by Dresing et al. (e.g., transcribing literally and not phonetically, translate dialects in standard language) [43]. All researchers, and especially the two researchers involved in the data collection (ER and EÖ) read the generated transcripts several times very carefully and if necessary the recordings were listened to again to ensure that no information was lost. The interviews were then pseudonymized by EÖ who replaced all names and places. We used MAXQDA 2018 for organizing the data [44].

The data analysis followed the steps according to qualitative content analysis by Mayring [45]. A category system was developed and discussed by all authors. The approach for developing categories was both deductive (derivation of content of the semi-structured interview guide considering the GDA framework [3]) and inductive (elaboration of additional themes in the material). To continuously revise the category system, a coding guide with illustrating quotes was created by ET and EÖ and discussed within all authors. Although the interview guide was mainly oriented to only three GDA themes (work organization, work content and task, and new forms of work), the content of the interviews and focus group interviews could be assigned to all five GDA themes. Therefore, the category system covers the following GDA themes: “Work content and task” (115 codes), “Organization of work” (191 codes), “Social relations” (21 codes), “Working environment” (16 codes), and “New forms of work” (14 codes). During our analysis, it became evident that the main category “Organization of work” prevailed in our material, and that other psychosocial aspects are highly interrelated to this category. So, the research team decided during the further process to focus on this main category, and we performed a more in-depth analysis within this category.

During the whole research process we applied quality criteria for qualitative research developed by Mays and Pope [46, 47]. Several rounds of analysis were carried out in tandem teams between ET, EÖ and AW to ensure intersubjectivity during data analysis. Due to time restrictions and for organizational reasons (the COVID-19 pandemic hit Germany just after the last interviews), it was not possible to reflect and validate the results back to the participants. We therefore discussed the methodological approach and the results in two separate meetings with four to six others health services researchers from our institute. After completing the analysis with first and second reduction [45], AW translated all quotes from German into English. Since no conversational analytical approach was used [48], and the research team focused rather on the overall content and meaning of the data collected, we expected no significant loss of meaning due to the translation.

## Results

This study investigates psychosocial demands related to work organization depicted by managers and employees in the health and service sector. During an in-depth analysis, we focused on the main category “Organization of work”. A complete overview of the subcategories and themes of this category is presented in Supplementary Material 3.

Within this category, the following four themes prevailed and were frequently reported by managers and employees: “Possibilities and time for recovery after work”, “Communication and cooperation with customers”, “Work intensity”, and “Interruptions and prioritization”. While these themes primarily address aspects of work organization, they also reflect and are closely interrelated to other themes of the GDA framework such as work content and tasks, and social relations. Thus, these four themes illustrate the various psychosocial demands faced by managers and employees in the health and service sector.

Subsequently, we describe the four themes in detail presenting the results assigned to the perspectives of managers and employees to highlight differences in the way work-related psychosocial demands related to work organization are perceived.

### Possibilities and time for recovery after work

#### Perspective of managers

As a result of work organization, managers from all companies reported various examples for a lack of possibilities and time for recovery after work due to limited division between work and private life. Managers had to organize many duties during and beyond the working hours of the company (e.g., work at the weekend, meetings with other managers, certain urgent administrative tasks, availability for urgent matters outside working hours).

*Manager, orthopaedic company: “For employees, if they call me,…if they write me something via WhatsApp or something like that, or at home on the private phone or on the mobile phone…that's fine for me. Or when we go on holiday, it's the same, then it's clear that if there's an emergency or something, you have to do something special, then they send me a picture and I write back in the evening.”*

These aspects of work organization (WhatsApp group, availability during holidays, …) are in turn closely related to aspects of work content and task. For example, the manager from the optician and hearing aid company reported that they often carried home perceived mental stress from work and reflected on work issues and possible solutions, since there is no time during work to think about such things. In the cosmetic company, managers often worked without breaks or with only few breaks between customers. In contrast, one manager of the optician and hearing aid company stated having regular breaks. Managers from two companies (dental technology company, cosmetic studio) also reported that they worked for very long hours. Working days were long in the cosmetic company in order to meet customer requirements (as customers in this setting mainly come to an appointment in the afternoon or evening).

*Manager, cosmetic studio: “We are not an industrial company. Many applicants for a job just say: Yes, I'll come from 7 a.m. to 4 p.m.. Then I say: Yes, OK. Nice fantasy. If I work at Mercedes, BMW and so on, that's possible. But that's not possible in a small company. So, we have to adapt to the customer. (…)”.*

During the interviews, there was hardly any discussion about the consequences of a lack of possibilities and time for recovery after work. One manager (orthopaedic shoemaker) reported paying attention to an appropriate level of recovery after work. This was related to a recent serious illness that resulted in a significant lifestyle change, which included a reduction of work-related duties through delegation of responsibilities. In another interview, permanent availability for work-related issues was not seen as a major problem (orthopaedic company). Nevertheless, managers seemed to be aware of the lack of possibilities and time for recovery after work and different coping strategies were therefore used to deal with this issue. One coping strategy aimed at maintaining stable working and business hours, and, if possible, to avoid working on weekends (pharmacy, orthopaedic shoemaker). Another manager stated that they tried to get things accomplished at the workplace, desire to limit the own availability to the opening hours, and during the regular working hours to keep a balanced workload with sufficient rest (orthopaedic shoemaker). In one family business (orthopaedic company), the possibilities and time for recovery was supported by the feedback of the children of the managers encouraging their parents to try to switch off from work at home. Another coping strategy was the hiring of additional staff in order, for example, to be able to further reduce the work as a spontaneous replacement for sickness absence of employees.

*Manager, pharmacy: “And then I'm often the person who jumps in when someone is sick or on holiday. And now I have reduced it again, because I have just hired a pharmacist and I am no longer so much at the front of the hand-selling.”*

#### Perspectives of employees

Compared with managers, employees reported fewer problems regarding the division between work and private life. Some examples for a lack of possibilities and time for recovery after work were courses for further education in leisure time (pharmacy), and the use of WhatsApp groups for work-related issues (pharmacy, optician and hearing aid company). The use of the work-related WhatsApp group was, however, not perceived as a burden (pharmacy).

Contrary to the managers, employees rarely worked overtime (orthopaedic shoemaker), and usually fulfilled their working time within their contractually agreed hours. Also, in most companies, breaks for employees were scheduled. Often, for example, the shop was closed for a certain period of time during lunch breaks, and during this time it was possible for employees to take a break.

*Employee, pharmacy: “We close from 12:30 to 14:00. And that is fine, too. The manager agrees and that's also good, just to come down for a while.”*

In certain companies (orthopaedic company, cosmetic studio), employees decided when they take a break, depending on their work schedule. In the optician and hearing aid company the shop did not close at lunchtime. Here, the employees agreed among themselves who will take a break and when. All employees stated that it rarely happens that employees cannot take their break and have to continue working. Since many companies were located in the middle of the city, breaks were used to get some fresh air.

*Employee, orthopaedic shoemaker: “Yes, that's actually … you get something, you go to a restaurant, you occasionally bring something with you. Whatever you want, whatever you feel like.”*

One employee from the orthopaedic shoemaker company (orthopaedic shoemaker) addressed furthermore some strategies for receiving a good level of recovery after work. He did not take work home, tried to resist stress and reduce overtime in agreement with his manager.

### Communication and cooperation with customers

#### Perspective of managers

According to the managers, several requirements were necessary for good communication and cooperation with customers. Here, as well, the link between aspects of work organization, work task and content as well as social relations emerges. With regards to work organization, disruptions of customers’ conversations or treatments should be avoided (optician and hearing aid company, cosmetic studio). For the managers, it was essential that customers are in the centre of attention (orthopaedic company) and that a good customer service is ensured (optician and hearing aid company, orthopaedic company). Other requirements were the consideration of good social relations in form of communication at the same level with the customer (dental technology company). Managers from two companies (orthopaedic company, cosmetic studio) mentioned in particular a close customer relationship and personal contacts. Furthermore, they stated that good communication has a strong impact on customer loyalty.

*Manager, cosmetic studio: “Rather, it's all about that, because customer loyalty is often present, i.e. very strong customer loyalty here. We have noticed that in the extreme (…).”*

This theme also revealed differences between the companies. While five companies (pharmacy, optician and hearing aid company, orthopaedic company, orthopaedic shoemaker, cosmetic studio) had a “classic customer base” with customers or patients entering the store, the dental technology company represented a unique case. For the dental technology company, the dentists were the customers, and the company received the work assignments from them. Thus, the customers of the dental technology company were also experts in the same field. Due to the special situation of the dental technology company, honesty, openness and willingness to criticize were considered by the manager as important requirements for good communication and cooperation with the dentists. They also emphasized several challenges regarding the communication and cooperation with them.

*Manager, dental technology company: “We are of course greatly dependent on the dentists. And this is always a big issue, because the dentists can of course send the work to another laboratory from now on. And this is a dependency that I don't like that much (…). I want to be part of the team, and actually we are, dentists and dental technicians, because both sides have to work together, and some dentists don't see that. They always think they are at the top because they are the client.”*

This dependency is often related to a high degree of economic pressure. Therefore, the manager of the dental company described his customers differentiated in terms of cooperation with them. Clients with whom the cooperation is perceived difficult, often do not adhere prior agreements. The communication and cooperation with dentists affect the whole work situation, and the motivation of the employees:

*Manager, dental technology company: “(…) Motivation of the dental technicians is enormously important, because if you only have customers who treat you from above, you don't like to work for them. And at some point, the quality you deliver is necessarily the same. And if you are subconsciously influenced by customers you like, with whom you can really exchange ideas, even in critical moments, it works much better (…).”*

#### Perspective of employees

Employees of the companies shared a similar way of looking at the theme communication and cooperation as the managers. Employees from the orthopaedic company regarded the following requirements as important: friendliness, competence, empathy, tolerance, taking time for the customer, and taking them seriously. Other requirements to facilitate communication and cooperation were to ensure good customer care (optician and hearing aid company, orthopaedic company), mutual respect (optician and hearing aid company), and the ability to learn how to deal with customers (orthopaedic shoemaker).

*Employee, orthopaedic shoemaker: “And that's where I learned most of it. How to deal with people. And also, when people come in that you can see from the beginning, like, what kind of guy, what's coming at you. It took a long time before it sat like that, but that's what I thought. But it works out quite well.”*

Employees of the dental technology company also cited challenges regarding the cooperation and communication with dentists as customers. Frequent challenges were an insufficient flow of information and the difficult availability, for example to make enquiries:

*Employee, dental technology company: “Yes, also from the information about the desired work, that's exactly the same, not everything is written on it, then you have to ask, you don't catch anybody. But the appointment is made ... and then the practice is closed again in the evening or at noon.”*

### Work intensity

#### Perspective of managers

Managers from different companies reported being confronted with high work intensity. Reasons for high work intensity for managers were an additional high bureaucratic workload, and the changing and not predictable number of customers (orthopaedic company), that the companies were confronted with. The orthopaedic company is also responsible for providing acute therapy for customers:

*Manager, orthopaedic company: “Of course. It is always a challenge for us when there are 20 people in the clinic and then ten come to us and need urgent care immediately, then we sometimes pile up the people. And we also need our time if a shoe needs to be changed immediately. This is a big challenge, which we have to face with our personnel, because these are all things that have to be changed immediately.”*

Managers tried to find solutions to deal with high work intensity. They try to improve the work organization by for example extending the processing time, or in some cases, the working time was also extended (orthopaedic shoemaker).

*Manager, orthopaedic shoemaker: “Or, what we do when things get really tough, which happens two or three times a year at most, we say: OK. We're usually closed on Wednesday, and the shop is closed and the repair shop is closed, but then we just work for a few hours (…).”*

Other solutions from the perspective of managers were to perform their administrative tasks only when there were a few customers in the shop (orthopaedic company). High work intensity resulted in perceived stress for managers as well as stress due to deadline pressure (orthopaedic shoemaker), and to waiting customers (orthopaedic company).

#### Perspective of employees

Employees provided additional insights into the theme work intensity. Reasons for high work intensity were bureaucratic factors and occurring delivery shortages (pharmacy), the changing and not predictable number of customers (optician and hearing aid company), an increase in incoming orders (orthopaedic shoemaker, cosmetic studio) or phone calls (optician and hearing aid company). Employees from two companies reported an increase in work intensityat certain times of the year, for example during the Christmas season for the dental technology company and in spring for orthopaedic mechanics:

*Employee, orthopaedic shoemaker: “When it's springtime now, people think, oh yes, now I should prepare myself for my spring shoes. The loafers, sandals, everything they have. And of course, they'll bring all that stuff. And then it can happen that you have quite a pile of work.”*

Other reasons for high work intensity were due to required corrections of job tasks, and repeated work steps and delays (dental technology company). Employees of the same company also indicated that work orders and the periods of time allowed for their processing were often unpredictable, external determined and short-term in nature.

*Employees, dental technology company: “Right. Yes, and it's generally the same with the deadlines, so I think that no matter who does what, it's somehow the same with everyone, because it's really a bit … You can't predict it. For example, we got two dental crowns today, so it's always … You can never tell, there can be something new in the morning, at noon, in the evening, so it's always. Exactly.”*

As a consequence of high work intensity, the most frequently mentioned factor for employees is also perceived work-related stress including stress due to completing increasing tasks (e.g., during springtime) (orthopaedic shoemaker), as well as due to the correction and repetition of job tasks (dental technology company). Another consequence for employees of the orthopaedic shoemaker and the dental technology company was that their own ability to plan their work suffers due to externally determined processing periods or specific customer demands.

*Employees, dental technology company: “And that's just the difficulty with us, the planning, a certain amount of planning is already possible, that's fine. But as I said, if something goes wrong in between, or if something goes wrong at the dentist, for example, and we have to repeat it again or have to repair it, or whatever, that's the difficulty for us. That is the stress, so to speak (…).”*

Employees of two companies (orthopaedic shoemaker, dental technology company) specified suggestions to avoid high work intensity. Suggestions included reasonable processing time, and better coordination of work steps so that they run more smoothly. Employees of the dental technology company also suggested more influence on time scheduling and good working documents with complete instructions to guarantee good work for the customer (in this case the dentist). They emphasized that the dentists as customers should prepare the required measurements to the dental laboratory (e.g., for dentures) as specifically as possible. According to the employees, this avoids and reduces subsequent corrections and repetition of job tasks, for which the dental technology company is not compensated.

### Interruptions and prioritization

#### Perspective of managers

Managers reported many interruptions during their working day. They indicated that the processing of administrative tasks and customer conversations were frequently interrupted (orthopaedic company). In the cosmetic studio, interruptions of ongoing tasks were reported when new customers entered the store while other customers were being treated and no additional staff members were available. In such cases, the treatment would be interrupted to serve the new customer. Yet, these interruptions interfere with the manager's instructions that the treatment and contact with customers should not be interrupted (cosmetic studio). However, incoming customers have a higher priority than incoming phone calls. In the cosmetic studio, customers were encouraged to leave their requests on the answering machine and make online reservations themselves because serving current customers in the store was understood as the priority task:

*Managers, cosmetic studio: “And that's why we said, all right, phone's ringing, let it ring. (…) Yes, but the customer who is in the shop has the priority, that's the highest priority.”*

Managers try to deal with interruptions including strategies such as the prioritization of specific work tasks (orthopaedic company, cosmetic studio) and the scheduling of certain work activities before opening hours:

*Managers, orthopaedic company: “I am a person who can do that, but you often don't get into focused work activities in such a way that you can really think your way into something that you can stay at it for a long time. That's actually already … Well, my life is here … really characterized by interruptions every day. The fact that I can stay at something for a quarter of an hour is rather the exception. That's why I love the early morning hours.”*

The most frequently perceived negative consequences of interruptions reported by managers were increased perceived stress, and a lack of concentration (orthopaedic company).

#### Perspectives of employees

Employees also listed many examples for interrupted work tasks, e.g. administrative tasks (optician and hearing aid company, orthopaedic company), tasks in the repair shop (optician and hearing aid company) and customer conversations (orthopaedic company). Employees explained why some of their work tasks were interrupted and other tasks were prioritized. The main reason for interruptions was due to additional customers who could not be served by additional personnel (optician and hearing aid company, orthopaedic company, orthopaedic shoemaker). Other reasons for interruptions in the different companies included incoming phone calls (optician and hearing aid company, orthopaedic company), equipment failure (orthopaedic shoemaker), queries from the manager (orthopaedic company), and the parallel running of store and repair shop (orthopaedic shoemaker).

*Employee, orthopaedic shoemaker: “(…) And now you are interrupted during this work. Yes, it's nothing new for me, because … I've been doing this job for about 30 years now and I've also worked with customers before. I have also been interrupted at times. So, it's nothing new and doesn't bother me more or less. Sometimes it's stressful, … sometimes you have more repairs and then there are more customers and then it all gets a bit annoying, a bit stressful.”*

In the pharmacy, some work tasks were very urgent and needed to be prioritized. In particular, acute care and preparation of refrigerated products were specific work tasks that were given a high priority. Employees of the dental technology company often had to interrupt their work process due to sudden arising deadlines for incoming new orders:

*Employees, dental technology company: “And every customer has different deadlines. One gives us two weeks to do a job, another gives us three weeks, and the third has to be done in a very short time, so you can only plan to a limited extent.”*

Employees used under supervision and in agreement with their managers different strategies to deal with interruptions of work tasks. Strategies included the prioritization of specific work tasks (optician and hearing aid company, orthopaedic company), creating reminder notes (pharmacy), attempting to rapidly complete most of a task (pharmacy, orthopaedic company), not answering incoming phone calls (orthopaedic company, cosmetic studio), or spontaneous coordination of work tasks with colleagues (optician and hearing aid company). In the optician and hearing aid company, work begins before the opening of the store in order to avoid interruptions during the dealing with repairs:

*Employees, optician and hearing aid company: “And I think that my colleague is now one hour earlier because of the repair shop, because there are many orders where only lenses are ordered (…) The daily business is still in the repair shop. And there are also many things that are perhaps a bit more extensive. And that you can perhaps … that you can perhaps do a bit of preparatory work in peace.”*

Many consequences were mentioned which are caused by interruptions at work. The most frequently mentioned consequences by employees were long processing times (optician and hearing aid company, orthopaedic company), a perceived disruption of the work routine (orthopaedic company), tension (pharmacy), perceived psychological stress (optician and hearing aid company, orthopaedic shoemaker), being annoyed (optician and hearing aid company), and mistakes.

*Employee, pharmacy: “Of course I try to keep everything organized, but if the product is not back where I put it, then I get a bit tense and then I think to myself: Where did I put it after all? Then, yes, then you have to do one thing at a time. I try to learn how to do it, because otherwise mistakes are made and then you are in a stressful situation.”*

## Discussion

In previous studies, managers and employees of SMEs in the health and service sector have been rarely considered. Our results provide an overview of work-related psychosocial demands associated with work organisation and work content typical for the health and service sector, comprising the themes “Possibilities and time for recovery after work”, “Communication and cooperation with customers”, “Work intensity”, and “Interruptions and prioritization”. Subsequently, we discuss our findings from six different SMEs in Germany with regard to the differing perspectives of managers and employees.

### Work-related psychosocial demands explored by managers and employees

#### Possibilities and time for recovery after work

Compared with their employees, managers were more likely to report issues related to a lack of possibilities and time for recovery after work. A major challenge was that managers were responsible for the company and for their teams. As well, the managers were usually the owners of the companies and were personally liable with their private wealth. The constant responsibility to run the company and to shape working conditions in such a way that employees are satisfied can hinder self-care, including sufficient leisure time and possibilities and time for recovery after work for managers. There are intervention studies aiming to enable managers in SMEs to incorporate team care and self-care, also promoting the prevention and reduction of psychosocial risks for the entire team [29, 49]. The results of the intervention studies imply that improved mental well-being of managers can enhance psychosocial working conditions and well-being of employees [49]. The harmful consequences of a lack of possibilities and time for recovery, constant availability and insufficient break time were hardly discussed by the managers interviewed. However, some studies indicate harmful effects on health, like problems with falling asleep and sleeping through, problems to relax and recover, exhaustion, stress, and nervousness [50–52]. Only one manager seemed to be aware of this topic due to a serious illness from which he previously suffered. Summarizing, we assume that managers from these settings might need additional support and advice in dealing with these demands to avoid harmful and unhealthy effects for their employees and themselves. Other studies have already demonstrated that SMEs need additional support and more infrastructure for dealing with these psychosocial demands [22, 23]. As outlined by Pundt and Felfe (2017), self-care is the basis for health-oriented leadership directly impacting the company owners’ ability to care for their staff and, in turn, promote health relevant behaviour at work [53]. Employees talked in the interviews less about work-related demands and more about resources they draw for themselves. They appeared to be less affected by a lack of possibilities and time for recovery or permanent availability for work-related demands. This may be due to the fact that they mainly worked within their scheduled working hours, within defined and regulated working conditions set by their managers, and without having the permanent responsibility for the entire company. This also reflects the high priority of the company owners included in our study to provide a good working environment for their staff. Nevertheless, excessive use of a WhatsApp group for work-related purposes beyond working hours should be viewed critically, as it can negatively affect private life and promotes, for example, poorer switching off from work and lower recovery values [51].

#### Communication and cooperation with customers

The theme “communication and cooperation with customers” revealed for most companies shared values between managers and employees about the importance of good communication and cooperation with customers. Within this theme, we identified a major difference between the companies. While five companies had a "classic" client relationship with a clear division of roles between customers and professional experts (e.g., customers and patients were coming to the orthopaedic store for consultation), the clients of the dental technology company (= dentist) were acting as intermediary for their own clients (= patients). This represented a rather different customer relationship with reversed hierarchies. Here, patients were only indirect customers and received the prepared product from the dental laboratory via the dentist. Both managers and employees of the dental technology company reported about several problems in the context of work organization regarding cooperation and communication with the dentists who acted as demanding co-experts and professional expertise overlapped. Additionally, the incomplete or imprecise specifications for dental products (e.g., dental crowns) given to the dental technology company by the dentists resulted in avoidable and additional cost for the company who carried the entire financial risk for providing perfectly fitting products for the patients of the dentists. This problem is also reported and well-known in several studies [54–59]. One main impediment to good communication was the lack of time reported by dentists [58] combined with a lack of financial risk regarding the consequences of providing the correct specifications for their orders. One study recommended that dental students should be trained to communicate effectively and provide all the information the dental technician needs [54]. Another study demonstrated a slight improvement of prescriptions and the reduction of errors through education and training as part of an audit [60]. Overall, it seems worthwhile to improve constantly communication and cooperation between these two professional groups in order to reduce stress and strain as well as avoidable economic loss for the dental technology companies.

### Work intensity

Both managers and employees in all companies were affected by high work intensity. In addition to customer service, a large number of bureaucratic tasks caused high work intensity. This administrative work was often postponed in order to complete prioritized tasks such as serving customers. However, postponing tasks resulted, in turn, in increased work intensity and probably even fewer breaks. Employees also indicated to be affected by high work intensity that usually occurred within specific periods of the year. A recent scoping review suggests that high work intensity can be managed through appropriate staffing, good task and break design, improved role clarity, transformation of the work environment, and the creation of further training opportunities to empower personal resources [61]. This requires managers to be aware of this issue and proactively take measures for themselves and employees to deal with high work intensity. In our sample, the employees of the dental technology companies were especially confronted with unexpected and unpredictable work, e.g. due to externally determined deadlines. This resulted in perceived stress and is difficult to prevent. One way is certainly to talk to the customer (in this case dentist) and make agreements for a good cooperation for both sides to avoid high work intensity among the employees. But this also reveals the difficulty of the dental technology company in demanding something from its customers, since they are economically dependent on them.

### Interruptions and prioritization

Managers and employees across all companies were confronted and exposed to frequent interruptions. The interviewed managers and employees dealt with the interruptions at work by postponing administrative tasks and prioritizing specific work activities, e.g. consulting for customers. The interviewed managers and employees stated having frequent interruptions lead to stress and mistakes. Previous studies showed that frequent interruptions of work activities can contribute to increased cortisol level as stress indicator, reduced job satisfaction and increased psychosomatic complaints [62]. The following approaches to avoid interruptions are currently discussed: setting up “no disturbance” signs, special training on work strategies for dealing with interruptions, a clear definition of roles within the team, proactive communication and the possibility of an uninterrupted work phase ("quiet hour") [62]. However, possible interventions to avoid interruptions have so far hardly been implemented and discussed in SMEs. Often these approaches are difficult to implement due to a lack of time and financial resources of SMEs [30]. Thus, a tense staff situation often leads to interruptions, for example, because a customer has to be served. Yet, further research is needed to identify appropriate approaches to deal with frequent interruptions in SMEs and establish support for company owners and managers to fulfil their leadership role, not only comprising a particular focus on staff care, but establish a health-oriented leadership style and culture beneficial for the entire company team [53].

### Strengths and limitations

This is one of the first qualitative studies exploring work-related demands for managers and employees in SMEs in the health and service sector. We focused thereby on results of the in-depth analysis of the main category “Organization of work” to comprehensively describe the identified themes and the differing views of managers and employees. As Schreibauer et al. described, a limited number of studies in SME settings have focused on psychosocial factors related to work content (*n* = 33), work organization (*n* = 31) or social relations (*n* = 26) mostly applying cross-sectional designs [21]. Thus, the use of a qualitative design helps to provide additional perspectives from managers and employees in SMEs. Overall, the inclusion of the six companies in the study has made it possible to cover the broad range of the health and service sector. The theoretical framework by the Joint German Occupational Safety and Health [3] and the analysis method according to Mayring (2015) were suitable for describing and structuring work-related demands from our interview material. Qualitative research is thereby very important here, as it can highlight individual perspectives of managers and employees in more detail. We pursued a high level of intersubjectivity through the continuous analysis in tandems. Further, the methodological approach and the results were discussed in two feedback meetings with other health services researchers, who were not involved in data collection or data analysis. Overall, this qualitative study can serve as a preliminary study for further studies focusing on SME settings in the health and service sector, as this sector has hardly been researched so far. For example, the results can be used when developing a questionnaire for the standardized assessment of sector-specific aspects of work organization as a starting point for tailored intervention offers in this setting.

Our study included, however, only a small sample of six different German companies in the services and health sector, which can be considered as a limitation. Therefore, no data saturation can be expected. In addition, it would have been difficult to conduct further face-to-face interviews after February 2020 in Germany due to the outbreak of the global COVID-19 pandemic. A communicative validation of our results with managers and employees of the six companies would have been helpful. However, due to time restrictions and other organisational reasons related to the pandemic, we were not able to perform this. In addition to the interviews and focus group discussions, we planned to conduct a participatory observation of team meetings to triangulate different methods but had to do without it as the companies didn´t perform regular team meetings. There are currently studies that also employ an observational perspective [25, 63] in order to provide a more comprehensive description of the research subject. Yet, this was not possible, so data from our interviews cannot be combined with additional data from observations regarding organization of work, and social interactions between managers and employees. Hence, a more complementary perspective is missing. Another limitation lies in our analysis process. Since we focused in this publication on the main category “Organization of work” of the GDA framework [3], the results of the other main categories (i.e. work content and tasks, social relations, working environment, and new forms of work) are still outstanding and will be subject of further publications to give more insights on psychosocial demands of people working in SMEs in the health and service sector. However, the results of the main category “Work organization” cannot be considered as distinct from other GDA categories. Rather, it was found that the selected topics were also accompanied by other psychosocial factors related to work content and task, and social relations. Thus, our results emphasise the complexity of the workplace. Concerning the cosmetics company, we only have the perspective of the two managers, as the employees withdrew from a focus group discussion at short notice. Here, unfortunately, the employees' perspective is missing and could be considered by future research.

## Conclusion

Our results show that managers and employees from the health and service sector experience various work-related demands related to work organization, but in different degrees. Managers were in particular confronted with a lack of possibilities and time for recovery after work. They also were confronted with high work intensity and frequent interruptions and prioritization, and report issues related to inadequate communication and cooperation affecting the entire company team. They use behavioural prevention measures (e.g., modification of personal factors), but measures for structural prevention (e.g., modification of workplace conditions) were taken less often. The risk assessment could help, for example, identifying psychosocial demands and stressors according to the STOP principle (= Substitution, Technological, Organisational, and Personal measures) [64] by using appropriate structural and behavioural prevention measures. Employees reported a clearer division between work and private life. However, they also face periods of high work intensity, frequent interruptions and the need for prioritization. In summary, it is evident that managers and employees in SMEs in the health and service sector would benefit from evidence-based and evaluated tailored interventions and approaches addressing managers and teams to improve work organization. Further research in this area is essential to shape stress-preventive working conditions, and to develop improved strategies and targeted approaches to improve work organization for managers and employees in SMEs in the health and service sector. Meanwhile, there are two currently ongoing research projects in Germany. One project of the Federal Institute for Occupational Safety and Health in Germany addresses active risk prevention of mental stress in different SMEs [65]. Another project is the PragmatiKK study which investigates activities to help different SMEs to prevent stress and develops support programs through an online platform [22, 66]. These projects may hopefully provide further insights on managing psychosocial demands and stressors in SMEs.

## Supplementary Information

**Additional file 1.**

**Additional file 2.**

**Additional file 3.**

## Data Availability

The datasets generated and analyzed during the current study are not publicly available due confidentiality reasons but are available from the corresponding author on reasonable request.
