# Supplementary material for: Work-related psychosocial demands related to work organization in small sized companies (SMEs) providing health-oriented services in Germany – a qualitative analysis
Source: BMC Public Health. 2022 Feb 24;22:390. doi: 10.1186/s12889-022-12700-4 (PMC8866918; doi:10.1186/s12889-022-12700-4)
Supplement: Supplementary file 2 — Additional file 2. [file 12889_2022_12700_MOESM2_ESM.docx]

1. **Interview guide for single interviews: managers and owners**

| **Topic 1: Work organization** |
| --- |
| **Introductory questions** |
| - As a manager/owner you have different tasks and responsibilities. Could you please describe your typical working day? - Where do you see particular challenges? - How work-intensive do you experience your working day? - What is important to you regarding the organization of work in your company? |
| **Questions about typical work processes** |
| - How is the cooperation with [colleagues and] employees in the company organised (e.g. responsibilities, work orders)? - What is important for you when working with [colleagues and] employees? - Which aspects are important to you as a team leader? - How do you organize sick leave? - How do you organize holidays? |
| **Question about digitization** |
| - What role do digital processes play in your everyday work? |
| **Questions about interaction/communication** |
| - What is important to you when you communicate with your team? - How do you organize your communication within the team? |
| **Questions on mutual support (team and supervisor)** |
| - How do you perceive the support of your team? (in the daily work/ in the organization of roster and holidays) - How do you deal with suggestions from your employees on how to organize the daily work routine? - How do you deal with personal/professional matters of your employees? |
| **Topic 2: Work task** |
| **Questions on work tasks** |
| - How do you decide who takes over which tasks in the team?   - Are there tasks that are only to be done by certain persons? Please describe them.   - Are there tasks that each team member performs? Please describe them.   - Are there overlapping areas of responsibility so that individual tasks cannot be explicitly assigned to a person? |
| **Questions about emotional demands** |
| - Are there any issues and challenges you are still dealing with after work? |
| **Topic 3: New forms of work** |
| **Questions on the general conditions of work design** |
| - What do you consider to be particularly important thinking about working conditions of employment?   - What is your perspective, for example, on (non-)fixed-term contracts?   - What is your opinion on different work time models?   - Can your employees receive regular training? How? - What do you consider to be particularly important thinking about working conditions in your company?   - What further training opportunities are available to you in your company?   - Did you e.g. have the opportunity to take part in training courses on management issues/ safety at work?   - How did you experience this?   - Are you available outside the working time (e.g. weekends) for company matters? |
| **Topic 4: Conclusion of the interview** |
| - What would you wish for to reduce stress? - Do you have any further comments on content that has not yet been addressed in the discussion but is important for your work? |
| **Thank you very much for your participation in this interview!** |

1. **Interview guide for focus group discussions/single interviews: employees**

| **Topic 1: Work organization** | |
| --- | --- |
| **Introductory questions – picture cards** | |
| - When you think of yourself and your role in the team, which of the laid out picture cards suits you? | |
| **Questions about typical work processes** | |
| - What are typical work processes from your daily work routine? Please describe them.   - What challenges are you facing in your daily work?   - How do you experience interruptions of activities, e.g. by colleagues, incoming customer calls or technical faults?   - How are breaks integrated into your daily work routine?   - How flexible are your working hours? | |
| **Questions about work intensity** | |
| - How work-intensive do you experience your working day? | |
| **Question about digitization** | |
| - What role do digital processes play in your everyday work? | |
| **Questions about interaction/communication** | |
| - What is important to you when you communicate with your team?   - employees among each other   - with supervisors/business partners/customers - How is communication in the Team organized?   - verbal, team meetings, regular, ad hoc, …   - in writing, by email, SMS, … - What is important to you when you communicate with customers/ business partners? | |
| **Questions on mutual support (team and supervisor)** | |
| - How do you support each other in the team (in your daily work/ in the organisation of rosters and holidays etc.)? - How does your manager support you? - Do you have ideas and suggestions how to improve work processes? How do you discuss suggestions and new ideas?   - within your team   - with your manager - How do you deal with personal/professional matters within the team? - How does your manager deal with personal/professional matters of you? | |
| **Topic 2: Work task** | |
| **Questions about the distribution of work** | |
| - How do you decide who takes over which tasks in the team?   - Are there tasks that are only to be done by certain persons? Please describe them.   - Are there tasks that each team member performs? Please describe them.   - Are there overlapping areas of responsibility so that individual tasks cannot be explicitly assigned to a person? - Are there work areas that you can only do together as a team? | |
| **Questions about emotional demands** | |
| - Are there any issues and challenges you are still dealing with after work? | |
| **Topic 3: New forms of work** | |
| **Questions on general conditions of work design** |  |
| - What do you particularly like about the working conditions of your employment?   - How do you assess your career prospects in the company?   - Are you satisfied with the general working conditions of your employment? (e.g. (un-) fixed-term contracts)   - Are you available outside the working time for company matters? - What training opportunities do you have in the company? | |
| **Topic 4: Conclusion of the interview** | |
| - What would you wish for to reduce stress? - Do you have any further comments on content that has not yet been addressed in the discussion but is important for your work? | |
| **Thank you very much for your participation in this focus group discussion/interview!** | |
