# Supplementary material for: Work-related psychosocial demands related to work organization in small sized companies (SMEs) providing health-oriented services in Germany – a qualitative analysis
Source: BMC Public Health. 2022 Feb 24;22:390. doi: 10.1186/s12889-022-12700-4 (PMC8866918; doi:10.1186/s12889-022-12700-4)
Supplement: Supplementary file 3 — Additional file 3. [file 12889_2022_12700_MOESM3_ESM.docx]

**Category system of the main category “Organization of work” including subcategories and themes**
